# Chemotherapy-induced immunogenic cell death in combination with ICIs: a brief review of mechanisms, clinical insights, and therapeutic implications

**DOI:** 10.3389/fphar.2025.1572195

**Published:** 2025-06-05

**Authors:** Chengwei Li, Xiaoyan Qi, Min Yan

**Affiliations:** ^1^ Department of Integrated Traditional Chinese and Western Medicine, Shandong Public Health Clinical Center, Shandong University, Jinan, China; ^2^ Department of Oncology, Zibo Central Hospital, Zibo, China; ^3^ Department of Respiratory Medicine, Shandong Public Health Clinical Center, Shandong University, Jinan, China

**Keywords:** cancer therapy, ICIS, chemotherapy, immunotherapy, clinical applications

## Abstract

The combination of chemotherapy and immune checkpoint inhibitors (ICIs) represents a promising strategy for enhancing the efficacy of tumor immunotherapy. This review elaborates on its mechanisms and clinical significances. Chemotherapy-induced immunogenic cell death (ICD) serves as the foundation of this therapeutic synergy, involving the release of damage-associated molecular patterns (DAMPs) such as calreticulin, ATP, and HMGB1, which enhance immune activation in the presence of ICIs. Clinical trials have demonstrated that this combination approach markedly improves clinical outcomes across multiple tumor types, including non-small cell lung cancer, melanoma, bladder cancer, and triple-negative breast cancer. In clinical practice, this combination is increasingly adopted as a first-line or advanced-stage treatment, often guided by personalized medicine approaches. However, several challenges persist, including the management of treatment-related toxicity, high costs, and the identification of predictive biomarkers.

## 1 Introduction

Tumor immunotherapy has fundamentally transformed the therapeutic landscape of cancer treatment (Yasinjan et al., 2023; Rui et al., 2023; da Silva et al., 2019). Immune checkpoint inhibitors (ICIs), including anti-PD-1 and anti-PD-L1 antibodies, have shown substantial effectiveness in certain patient populations (Dall’Olio et al., 2022; Naimi et al., 2022). These agents function by blocking inhibitory signals on T cells, thereby enhancing the immune system’s ability to target and eliminate tumor cells. However, not all patients respond favorably, and resistance to ICIs continues to pose a significant clinical challenge (Sun and Xu, 2020; Sharma and Allison, 2015; Hughes et al., 2016).

Chemotherapy, a longstanding cornerstone in cancer treatment, utilizes various drugs that primarily target rapidly proliferating cells, including cancer cells (Knezevic and Clarke, 2020; Jiang et al., 2024). These agents exert their effects by inducing DNA damage (Bai et al., 2024), arresting cell cycle progression (Sun et al., 2021a), and triggering cell death. Specifically, chemotherapy-induced ICD serves as the pivotal process enabling the immune system to recognize and attack tumor cells more effectively when combined with ICIs. ICD is marked by the exposure and release of immunostimulatory signals—particularly calreticulin, ATP, and HMGB1—which collectively enhance T cell-mediated immune responses (Bian et al., 2022; Obeid et al., 2007; Kroemer et al., 2013).

The synergistic potential of combining chemotherapy and ICIs is promising. This dual approach can amplify the immune system’s antitumor response (Qian et al., 2022; Galluzzi et al., 2020). Chemotherapy triggers ICD, leading to tumor antigen release and TME modulation, which subsequently activates antigen-presenting cells and promotes T cells recruitment to the tumor site. ICIs can block T cells’ inhibitory signals, further boosting their antitumor efficiency (Zouein et al., 2022). This combinatorialstrategy helps overcome the limitations of monotherapies, providing a more comprehensive and potent anticancer approach. It holds the promise of improved patient outcomes, particularly for those who do not respond adequately to either ICIs or chemotherapy alone (Roskoski, 2024; Li et al., 2022).

In addition to inducing immunogenic cell death, chemotherapy exerts multiple immunomodulatory effects within the tumor microenvironment. For instance, chemotherapeutic agents like cyclophosphamide and gemcitabine have been demonstrated to selectively deplete immunosuppressive cells, including regulatory T cells (Tregs) and myeloid-derived suppressor cells (MDSCs). This depletion alleviats local immune suppression and promoting effector T-cell infiltration and activity. Moreover, chemotherapy can enhance antigen presentation by upregulating major histocompatibility complex (MHC) class I molecules on tumor cells, thus improving tumor recognition by cytotoxic T lymphocytes (CTLs) (Galluzzi et al., 2015). These multifaceted immunological alterations, when combined with ICIs, foster a more permissive immune landscape that significantly enhances antitumor efficacy relative to monotherapy (Pfirschke et al., 2016).

## 2 Clinical trial outcomes of the combination of chemotherapy and ICIs in tumor immunotherapy

### 2.1 Melanoma

In the treatment of melanoma, combining chemotherapy with ICIs has emerged as a promising strategy. The CheckMate 067 trial compared the effects of ipilimumab and nivolumab combination therapy to ipilimumab or nivolumab alone in patients with melanoma (Wan et al., 2021; Choueiri Toni et al., 2023; Owonikoko et al., 2021). The results were noteworthy, with the combination regimen yielding the highest response rate and the longest OS observed. The median OS didn’t achieve the combination group, while patients receiving ipilimumab alone had a median OS of 19.9 months and 36.9 months in the nivolumab-alone group. These findings indicate that the combination of ipilimumab and nivolumab exerts a synergistic effect, intensifying the antitumor immune response and substantially improving survival outcomes.

In addition, pembrolizumab combined with chemotherapy has been tested in melanoma patients. A phase II trial examined the use of pembrolizumab alongside dacarbazine in metastatic melanoma patients. The outcomes suggest that this combination achieved a 56% response rate, with a median OS of 23.5 months and a median PFS of 8.9 months. These results suggest that integratingchemotherapy with ICIs may represent an effective therapeutic approach for melanoma, capable of improving both response rates and survival.

### 2.2 Bladder cancer

In the treatment of bladder cancer, combining chemotherapy with ICIs has shown notable advantages. The IMvigor130 trial investigated the addition of atezolizumab to chemotherapy (cisplatin and gemcitabine) vs. chemotherapy alone in patients with metastatic or locally advanced urothelial carcinoma (Funt et al., 2022; Galsky et al., 2024; Balar et al., 2017). The results showed a notable improvement in OS and FPS among patients with PD-L1-positive tumors. The combination therapy group experienced a median PFS of 8.2 months, while the chemotherapy-only group had a median PFS of 6.3 months. Additionally, the 12-month OS rate was 71% in the combination group, compared to 62% in those treated with chemotherapy alone.

The KEYNOTE-361 trial trial assessed the efficacy of pembrolizumab combined with chemotherapy (gemcitabine or docetaxel plus carboplatin or cisplatin) compared to chemotherapy alone in patients with urothelial carcinoma. While the combination therapy did not meet its primary endpoint for OS, it showed a positive trend toward improvoved PFS and response rates (Suzuki et al., 2023; Kelley et al., 2023; Sharma et al., 2024; Nakamura et al., 2023).

### 2.3 Triple-negative breast cancer

In triple-negative breast cancer (TNBC), the combination of chemotherapy and ICIs has produced encouraging results. For instance, the KEYNOTE-522 trial assessed the use of pembrolizumab combined with chemotherapy (carboplatin and paclitaxel followed by epirubicin or doxorubicin and cyclophosphamide) vs. chemotherapy alone in early-stage TNBC patients (Rizzo et al., 2022; Pusztai et al., 2024; Dent et al., 2024; Zhao et al., 2023). The trial demonstrated significant improvements in both event-free survival (EFS) and pathological complete response (pCR) for the combination treatment. Specifically, patients receiving the combination therapy achieved an 18-month event-free survival (EFS) rate of 91%, significantly higher than the 85% observed in those treated with chemotherapy alone. Additionally, the combination group achieved a pCR rate of 65%, which was higher than the 51% observed with chemotherapy alone. These results suggest that incorporating ICIs into chemotherapy could enhance outcomes for patients with TNBC, potentially lowering recurrence rates and boosting survival. This combination approach may offer a promising strategy for patients with this aggressive breast cancer subtype. A summary of key clinical trials is presented in Table 1.

**TABLE 1:** Clinical trial outcomes of combination chemotherapy and ICIs in tumor immunotherapy.

Cancer type	Trial	Combination	Outcome
Melanoma	CheckMate 067	Ipilimumab + Nivolumab vs. Ipilimumab or Nivolumab alone	Highest response rate and extended OS
Melanoma	Phase II Pembrolizumab Trial	Pembrolizumab + Dacarbazine	56% response rate, median OS 23.5 months, median PFS 8.9 months
Bladder Cancer	IMvigor130	Atezolizumab + Cisplatin/Gemcitabine	Improved OS and PFS in PD-L1-positive tumors; 12-month OS rate of 71%
Bladder Cancer	KEYNOTE-361	Pembrolizumab + Chemotherapy (Gemcitabine/Docetaxel + Carboplatin/Cisplatin)	Positive trend in improving PFS and response rates
Triple-Negative Breast Cancer	KEYNOTE-522	Pembrolizumab + Chemotherapy (Carboplatin/Paclitaxel + Epirubicin/Doxorubicin)	Improved EFS and pCR, 91% EFS at 18 months
Non-Small Cell Lung Cancer	KEYNOTE-189	Pembrolizumab + Platinum-based agent + Pemetrexed	Median PFS 8.8 months vs. 4.9 months (chemotherapy only), 12-month OS 69.2% vs. 49.4%
Non-Small Cell Lung Cancer	IMpower130	Atezolizumab + Nab-paclitaxel + Carboplatin	Improved OS and PFS, 18-month OS 64% vs. 52%, median PFS 7 months vs. 5.5 months
Head and Neck Cancer	KEYNOTE-048	Pembrolizumab + Chemotherapy (Cisplatin/Carboplatin + Fluorouracil)	Improved OS in PD-L1-positive tumors
Gastric and Esophageal Cancer	KEYNOTE-590	Pembrolizumab + Chemotherapy (Fluorouracil + Cisplatin)	Improved PFS and OS compared to chemotherapy alone

### 2.4 Non-small cell lung cancer

In non-small cell lung cancer (NSCLC), combining chemotherapy with ICIs has led to significant improvements in patient outcomes. The KEYNOTE-189 trial evaluated pembrolizumab in combination with chemotherapy (a platinum-based agent and pemetrexed) versus chemotherapy alone in patients with metastatic nonsquamous NSCLC (Gandhi et al., 2018; Wu et al., 2022; Yang et al., 2022; Huang et al., 2024). The findings were compelling, showing a marked improvement in progression-free survival (PFS) among patients receiving the combination therapy. In the combination group, the median PFS in the combination group was 8.8 months, significantly longer than the 4.9 months observed in the chemotherapy-alone group. This data highlights a significant delay in disease progression and offering patients more time with stable disease and improved quality of life. Additionally, the overall survival (OS) benefit was impressive, with 12-month OS rates of 69.2% in the combination group vs. 49.4% in the chemotherapy-alone group. These results indicate not only delayed disease progression but also a clinically meaningful extension in overall survival.

The IMpower130 trial further confirmed the efficacy of this combination in NSCLC. This trial compared atezolizumab combined with chemotherapy (nab-paclitaxel and carboplatin) to chemotherapy alone for the treatment of advanced nonsquamous NSCLC (Zhang et al., 2023; Felip et al., 2021; Horn et al., 2018; Park et al., 2023). The oresults confirmed that the combination therapy significantly enhanced OS and PFS. The 18-month OS rate was 64% in the combination set vs. 52% in the chemotherapy-alone set, while the median PFS was 7 months in the combination set, vs. 5.5 months in the chemotherapy-only set, while.

### 2.5 Other tumor types

The combination of ICIs and chemotherapy has also been investigated in several other malignancies, including head and neck, gastric, and esophageal cancers. In head and neck cancer, the KEYNOTE-048 trial assessed the effect of pembrolizumab combined with chemotherapy (cisplatin or carboplatin plus fluorouracil) in comparison to chemotherapy alone or with cetuximab in patients with recurrent or metastatic head and neck squamous cell carcinoma (Burtness et al., 2022; Harrington et al., 2022; Burtness et al., 2019). The study demonstrated that the addition of pembrolizumab to chemotherapy significantly improved OS in patients with PD-L1-positive tumors compared to chemotherapy alone.

Similarly, the KEYNOTE-590 trial evaluated pembrolizumab combined with chemotherapy (fluorouracil and cisplatin) versus chemotherapy alone in patients with gastroesophageal junction or esophageal cancer (Kojima et al., 2022; Sun et al., 2021b; Kato et al., 2019). This combination treatment significantly enhanced both PFS and OS compared to chemotherapy alone.

### 2.6 Real-world applications

For certain malignancies, such as NSCLC, the combination of pembrolizumab and chemotherapy has been established as a standard first-line treatment. This regimen has demonstrated superior PFS and OS compared to chemotherapy alone (Gandhi et al., 2018). Similarly, in melanoma, the combination of chemotherapy with ICIs such as ipilimumab and nivolumab may also be incorporated into treatment regimen (Larkin et al., 2015). For patients with advanced-stage cancers, particularly those with limited treatment options and poor prognoses after prior therapies, combining chemotherapy with ICIs may improve quality of life and delay disease progression. For instance, in TNBC and bladder cancer, the addition of pembrolizumab or atezolizumab to chemotherapy has shown promising benefits for patients (Emens et al., 2021).

In clinical practice, there is an increasing emphasis on personalized medicine approach. Physicians increasingly tailor treatment strategies according to tumor-specific characteristics, including biomarker expression profiles. Tumors with high PD-L1 expression may respond better to the combination therapy, while those with lower expression may require alternative strategies. Genomic profiling plays a crucial role in identifying mutations or alterations that may be more responsive to this combined treatment, thereby allowing for a more individualized and potentially more effective therapeutic approach (Xu et al., 2024; Malone et al., 2020).

### 2.7 Selected failed or negative trials

Although numerous clinical trials have demonstrated the efficacy of chemotherapy combined with ICIs, several studies have reported limited or negligible benefits. For instance, the KEYNOTE-361 trial evaluated pembrolizumab in combination with chemotherapy versus chemotherapy alone in advanced urothelial carcinoma. Despite showing a trend toward improved PFS, it failed to meet the primary endpoints for OS or PFS statistically (Powles et al., 2021). Potential reasons for this failure include a heterogeneous patient population with variable PD-L1 expression, suboptimal selection of chemotherapy agents for immune synergy, and insufficient biomarker-based stratification.

Another example is the IMvigor211 study in metastatic urothelial cancer, where atezolizumab failed to demonstrate OS superiority compared to chemotherapy in patients with high PD-L1 expression (Powles et al., 2018). Although early-phase trials yielded promising results, phase III studies failed to replicate these benefits, underscoring the variability of immune responses and the critical need for improved patient selection strategies. These failed trials highlight the importance of biomarker-guided patient selection, appropriate chemotherapy pairing, and understanding of tumor immunobiology to enhance future trial success. It is worth noting that the chemotherapeutic agents used in these successful combinations are recognized for their ability to robustly induce ICD, significantly contributing to the observed clinical benefits.

## 3 Analysis of clinical trial success factors

A comparative analysis of clinical trials highlights notable similarities and differences in treatment outcomes but alsoreveals several critical factors underlying the varying degrees of success observed when combining chemotherapy with immune checkpoint inhibitors (ICIs).

### 3.1 Commonalities and individualities across tumor types

Clinical trials spanning diverse cancer types (e.g., NSCLC, melanoma, bladder cancer, TNBC) consistently demonstrate that high PD-L1 expression is positively associated with superior clinical outcomes, as evidenced by studies such as KEYNOTE-189 and IMvigor130. Nevertheless, tumor-intrinsic characteristics significantly influence the therapeutic benefit. Melanoma and NSCLC typically exhibit more robust responses to chemotherapy-ICI combinations, This heightened responsiveness is likely attributable to their relatively higher tumor mutational burdens (TMB) and intrinsic immunogenicity, which enhance the potential for immune recognition and attack. In sharp contrast, bladder cancer demonstrates variable responses to such combinations. This variability indicates that the complexity and heterogeneity of the tumor microenvironment may profoundly impact the tumor’s responsiveness to chemotherapy-ICI regimens, as illustrated by the findings of KEYNOTE-361 (Reck et al., 2016; Galsky et al., 2024).

### 3.2 Why some treatments worked and others did not

Successful clinical trials, including KEYNOTE-189 (NSCLC) and KEYNOTE-522 (TNBC) typically employed regimens integrating chemotherapy agents with proven immunogenic potential (e.g., platinum compounds, taxanes). These regimens were highly effective in enhancing antigen presentation, depleting immunosuppressive populations (e.g., Tregs, MDSCs), and robustly inducing ICD, thereby potentiating the effect of ICIs. Conversely, trials with limited success or failures—such as KEYNOTE-361 in bladder cancer—commonly exhibited inadequate patient stratification, suboptimal chemotherapy drug selection, or less favorable immune modulation. These included insufficient patient stratification, suboptimal selection of chemotherapy drugs, and ineffective immune modulation. Such deficiencies impeded the creation of an optimal immune microenvironment, which is essential for the successful activity of ICIs (Gandhi et al., 2018; Felip et al., 2021; Pusztai et al., 2024).

### 3.3 Factors influencing superior outcomes

The selection of chemotherapy selection plays a pivotal role in determining treatment efficacy. Platinum-based chemotherapy regimens (KEYNOTE-189, IMpower130) consistently yield improved clinical outcomes due to their potent immunomodulatory effects, including robust ICD induction and enhanced CTL infiltration into tumors. Timing and dosing are equally critical: administering chemotherapy concurrently with or shortly before ICIs maximizes immune priming and antigen release, fosteringa favorable immune environment that significantly enhances therapeutic efficacy. A key determinant of success in chemotherapy-ICI combinations is the ability of chemotherapeutic agents to induce ICD. Agents such as anthracyclines, oxaliplatin, and cyclophosphamide are well documented to elicit strong ICD responses (Zitvogel et al., 2010). The combination of pembrolizumab and certain chemotherapy drugs (KEYNOTE-361) in bladder cancer illustrates such a scenario, indicating that not all chemotherapy agents synergize equally well with ICIs (Galluzzi et al., 2015; Pfirschke et al., 2016).

## 4 Mechanisms of action

The synergistic effect of chemotherapy and ICIs relies on a multifaceted mechanism network that collectively boost the antitumor immune response. As a cornerstone of cancer treatment, chemotherapy influences the immune system through various pathways. A pivotal mechanism is the induction of immunogenic cell death: chemotherapy-induced damage to cancer cells leads to the release of tumor antigens and DAMPs (Galluzzi et al., 2017; Kroemer et al., 2022). Tumor antigens are captured and processed by antigen-presenting cells (APCs), while DAMPs act as “danger signals” that alert the immune system. This dual activation primes APCs to mature and migrate to lymph nodes, where they initiate a cascade of immune responses—including the activation of CTLs—to recognize and eliminate tumor cells. Moreover, chemotherapy can modify the TME, which is inherentlyimmunosuppressive, filled with factors that dampen immune cell activity. A core fundamental mechanism driving the efficacy of chemotherapy in this combination is its ability to induce ICD, characterized by the release of DAMPs—including calreticulin, ATP, and HMGB1. Calreticulin promotes dendritic cell phagocytosis of dying cancer cells, ATP serves as a chemoattractant and immunomodulator for dendritic cells, and HMGB1 enhances antigen presentation and T-cell priming (Szulc and Woźniak, 2024; Li et al., 2024; Zhou et al., 2019; Kepp et al., 2014). By reducing immunosuppressive elements and releasing these immunostimulatory signals, chemotherapy transforms the TME into a more “inflammatory” state, thereby enhancing the responsiveness of ICIs and enabling a robust antitumor immune response.

ICIs are pivotal in amplifying the antitumor immune response. T cells express inhibitory receptorslike PD-1 on their surface, while tumor cells in the TME (tumor microenvironment) often overexpress ligands such as PD-L1 (Wei et al., 2018; Kuzume et al., 2020). When PD-L1 on tumor cells interacts with PD-1 on T cells, it transmitsan inhibitory signal that suppresses T cell activity, suppressing effective tumor attack. ICIs, such as anti-PD-L1 or anti-PD-1 antibodies, block this interaction, essentially releasing the inhibitory “brakes” on T cells, enabling them to restore their antitumor activity.

**FIGURE 1:**
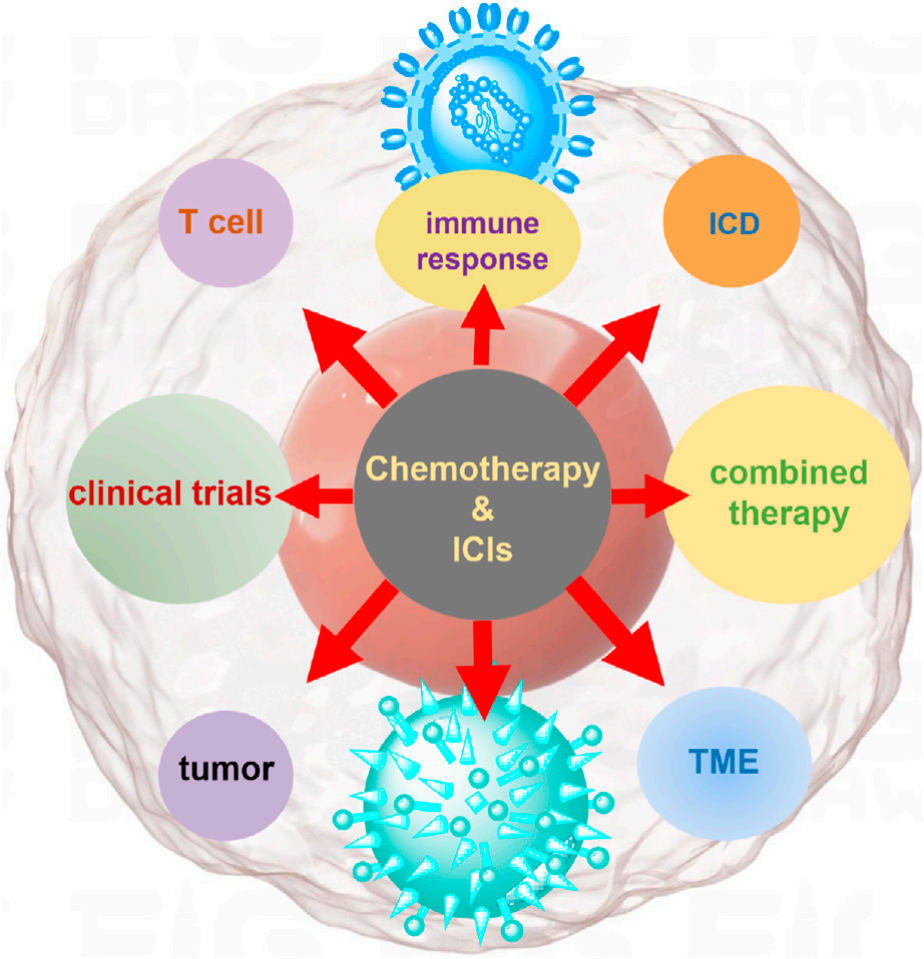
Mechanistic illustration of chemotherapy in conjunction with ICIs for tumor treatment. Chemotherapy induces ICD, leading to the release of tumor-associated antigens and DAMPs, such as ATP, calreticulin, and HMGB1. These signals activate dendritic cells (DCs), which process and present tumor antigens to CD8^+^ T cells. Chemotherapy also modulates the tumor microenvironment by reducing immunosuppressive populations such as Tregs and MDSCs. Meanwhile, immune checkpoint inhibitors (e.g., anti-PD-1, anti-PD-L1) block inhibitory signals between tumor cells and T cells, restoring T cell cytotoxic function. The combination of chemotherapy and ICIs enhances T cell activation, tumor infiltration, and tumor cell killing, providing a synergistic anti-tumor immune response.

When used in combination, ICIs and chemotherapy work synergistically (Hodi et al., 2010; Peggs and Quezada, 2010). Chemotherapy initiates a cascade by inducing ICD and alters the TME, thereby creating favorable conditions for immune activation. The activation of APCs and the release of tumor antigens prime naïve T cells and recruit them to the tumor site. Concurrently, ICIs prevent these T cells from being inhibited by the immune evasion strategies employed by tumors, allowing them to mount a stronger attack against tumor cells. This integrated approach offers a promising strategy to overcome the limitations of each treatment on its own and enhance the overall effectiveness of cancer therapy (Brahmer et al., 2015; Horn et al., 2017). As illustrated in Figure 1, the synergistic mechanism of chemotherapy-induced ICD in combination with ICIs. By promoting the exposure and release of damage-associated molecular patterns (DAMPs)—such as calreticulin, ATP, and HMGB1—chemotherapy enhances dendritic cell (DC) activation and T cell priming. This process creates a highly immunogenic TME that, when paired with ICIs, amplifies the anti-tumor immune response through sustained T cell-mediated tumor elimination.

## 5 Conclusion

In conclusion, the combination of chemotherapy and ICIs represents a highly promising strategy in tumor treatment. Mechanistically, chemotherapy induces immunogenic cell death and reprograms the tumor microenvironment, while ICIs block inhibitory signals on T cells, working synergistically to enhance the antitumor immune response. Clinical trials across diverse tumor types, including NSCLC, melanoma, bladder cancer, and TNBC, have demonstrated improved patient outcomes such as enhanced progression - free survival and overall survival. In clinical practice, this combination is increasingly utilized as a first-line or advanced treatment option, with a growing emphasis on personalized medicine. However, several challenges warrant attention, including toxicity management, cost considerations, and the identification of predictive biomarkers to guide patient selection. Future research should prioritize optimizing treatment protocols to further enhance the efficacy and safety of this combination, ultimately providing better treatment options for cancer patients. The analysis of unsuccessful trials underscore the necessity for meticulous trial design, including patient selection based on predictive biomarkers, appropriate chemotherapy regimens that promote ICD, and strategic treatment sequencing. These elements are indispensable for optimizing the therapeutic potential of chemotherapy and ICI combinations.
